# Development of a Knowledge-based Clinical Decision Support System for Multiple Sclerosis Diagnosis

**DOI:** 10.25122/jml-2020-0182

**Published:** 2020

**Authors:** Azamossadat Hosseini, Farkhondeh Asadi, Leila Akramian Arani

**Affiliations:** 1. Department of Health Information Technology and Management, School of Allied Medical Sciences, Shahid Beheshti University of Medical Sciences, Tehran, Iran

**Keywords:** Clinical, decision support system, diagnosis, multiple sclerosis

## Abstract

The diagnosis of multiple sclerosis (MS) is difficult considering its complexity, variety in signs and symptoms, and its similarity to the signs and symptoms of other neurological diseases. The purpose of this study is to design and develop a clinical decision support system (CDSS) to help physicians diagnose MS with a relapsing-remitting phenotype. The CDSS software was developed in four stages: requirement analysis, system design, system development, and system evaluation. The Rational Rose and SQL Server were used to design the object-oriented conceptual model and develop the database. The C sharp programming language and the Visual Studio programming environment were used to develop the software. To evaluate the efficiency and applicability of the software, the data of 130 medical records of patients aged 20 to 40 between 2017 and 2019 were used along with the Nilsson standard questionnaire. SPSS Statistics was also used to analyze the data. For MS diagnosis, CDSS had a sensitivity, specificity and accuracy of 1, 0.97 and 0.99, respectively, and the area under the ROC curve was 0.98. The agreement rate of kappa coefficient (κ) between software diagnosis and physician’s diagnosis was 0.98. The average score of software users was 98.33%, 96.65%, and 96.9% regarding the ease of learning, memorability, and satisfaction, respectively. Therefore, the applicability of the CDSS for MS diagnosis was confirmed by the neurologists. The evaluation findings show that CDSS can help physicians in the accurate and timely diagnosis of MS by using the rule-based method.

## Introduction

Multiple sclerosis (MS) is an inflammatory disease of the central nervous system [1-2]. It usually affects people aged 20 to 40 years [3-5]. MS is diagnosed on the basis of neurological history and physical examination, as well as the results of magnetic resonance imaging (MRI), evoked potentials (EP), and cerebrospinal fluid (CSF) analysis [6, 7]. Common diagnostic methods of this disease have problems and complexities, including

1. The diversity, instability and vagueness of the signs and symptoms [1, 5, 8, 9];
2. Its differential diagnosis from other diseases such as acute disseminated encephalomyelitis (ADEM) and neuromyelitis optica (NMO), and small vessel ischemic disease (SVID) [10-16];
3. The absence of any definitive diagnostic tests for it [1-5];
4. Limited specificity and accuracy of laboratory methods [1, 7, 12, 15], and limited access to diagnostic tools and procedures, as well as specialists in different geographic regions [17, 18].

Moreover, there is a significant delay from the stage of awareness of the initial signs and symptoms of MS to its diagnosis [10, 14, 18], causing irreversible injuries and disabilities [19-21]. Misdiagnosis is common in the case of this disease. It causes mistreatment and inappropriate use of medication, increases the severity of disabilities, mental and physical harm, incorrect diagnosis, ethical and medical challenges, and financial losses [20-24].

In recent years, intelligent systems such as the clinical decision support system (CDSS) have been used to solve many complicated issues [25-27]. These systems, in the form of different decision models, can help identify and diagnose diseases [28, 29]. Clinical knowledge in the knowledge-based CDSSs is often experimental and heuristic, derived from experts and guidelines and presented as a set of production rules [26, 30, 31].

Many researchers use software techniques to diagnose diseases and disorders of the nervous system [32, 33]. Various software systems such as the "Neurological Disorder Diagnosis System ", "Expert System for Diagnosis Neurodegenerative Diseases", and "Rule-Based Expert System for Diagnosis of Neuromuscular Disorders" have been developed for the diagnosis of neuromuscular disorders, which use clinical signs and symptoms to help users in the decision-making process and diagnosis of diseases of the nervous system, including MS [33-39]. They can help facilitate diagnosis and treatment, increase reliability and accuracy of diagnosis, reduce waste of time and expenses, increase access to neurologist's experiences, trains novice physicians and clinicians, indulge researchers in studies of neurological diseases and finally improve the quality of the healthcare system and quality of life of patients [33, 35, 39].

The purpose of this study is to develop a CDSS software and evaluate it in the case of relapsing-remitting MS to accelerate and improve the diagnosis of this disease.

## Material and Methods

**Figure 1:**
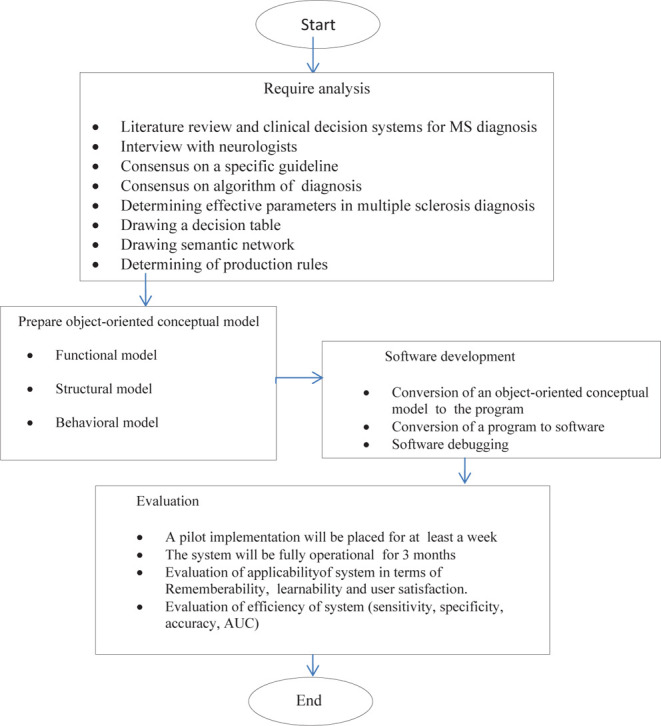
Developing Methodology of proposed system for MS diagnosis.

The software was developed in four stages, as shown in Figure 1.

1. Extraction of the requirements of system users: At the requirements analysis stage, data requirements, functional and non-functional requirements were determined in interviewers with 10 expert neurologists based on a semi-structured questionnaire. The questionnaire's validity was evaluated through its content validity, and its reliability was determined using the test-retest method (r = 0.87);
2. Software design: The software was designed in two stages, including designing the software's knowledge base and designing an object-oriented conceptual model.

### A. Designing the knowledge base

**Figure 2.**
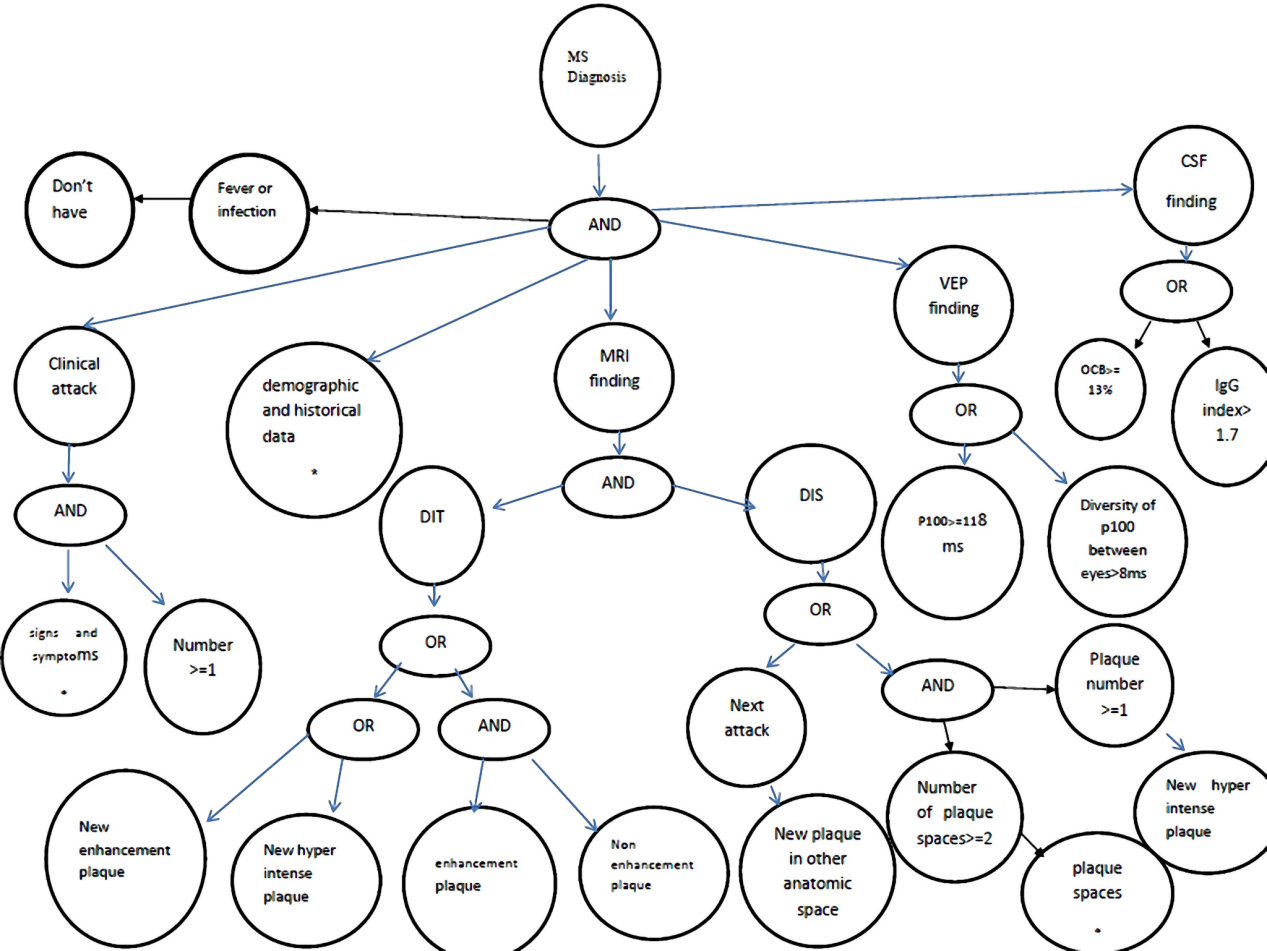
Level one of the semantic network of MS diagnosis.

**Figure 3.**
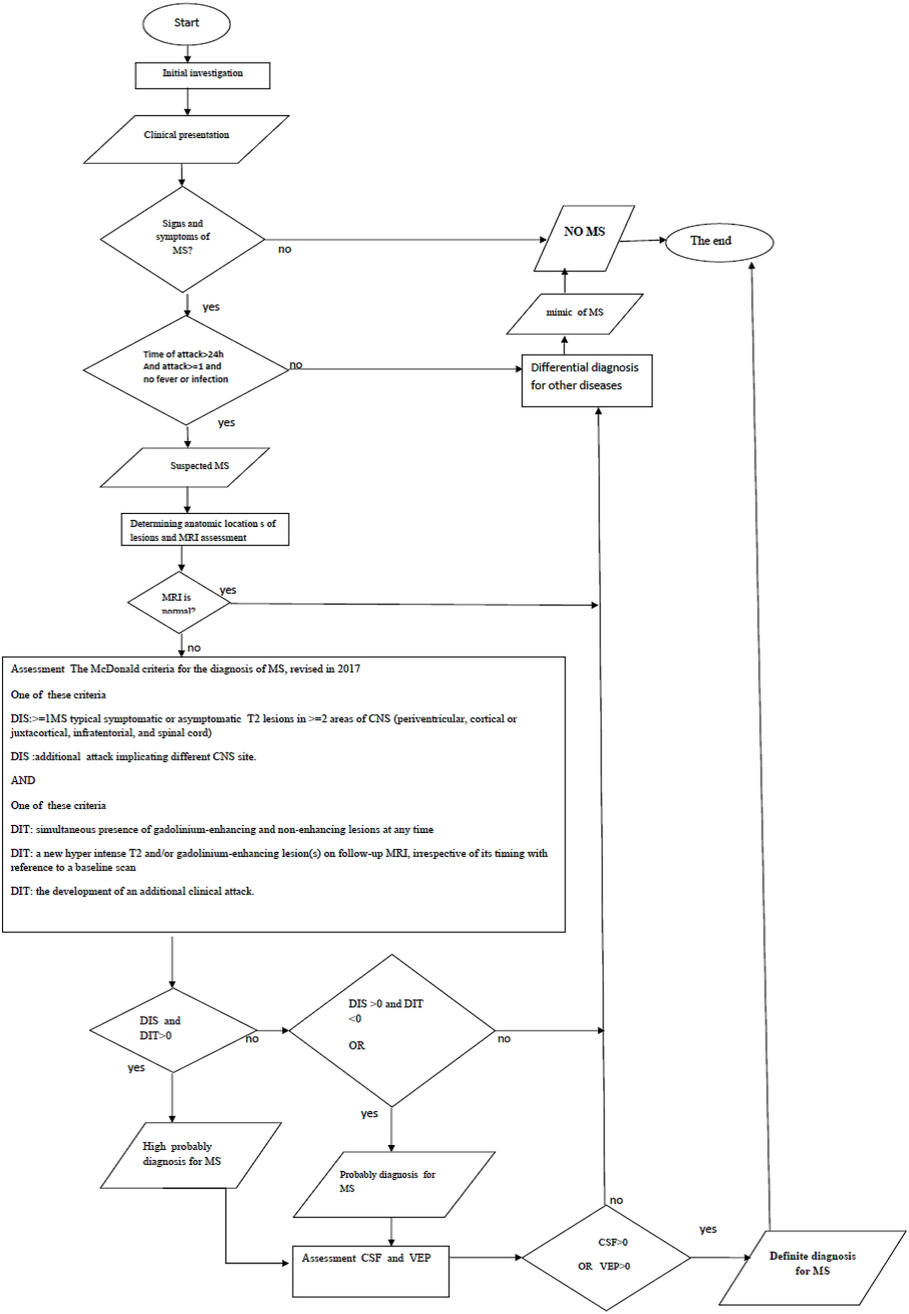
Multiple sclerosis diagnosis flowchart.

In order to design a knowledge base, the following should be conducted:

• Knowledge acquisition: Acquiring medical knowledge and identifying important and effective parameters regarding the diagnosis of MS occurs in two steps. In the first step, books, specialized texts, and articles concerning central nervous system diseases, MS diagnostic criteria, and MS diagnosis guidelines are studied, followed by identifying clinical signs and symptoms of MS diagnosis. In the second step, targeted interviews are conducted with 10 expert neurologists to determine the effective parameters in the diagnosis of MS, the relationship between clinical decision-making parameters and MS, the incidence of clinical signs, and the relationship between clinical signs and symptoms and the location of plaques in the central nervous system.
• Knowledge representation: The decision tables (Tables 2 and 3) and the semantic network (Figure 2) are used to organize and represent the CDSS knowledge. Decision tables and semantic networks show the relationship between the symptoms of the disease and the location of plaques in the central nervous system, as well as the relationship between decision parameters (clinical signs and symptoms and test results) and MS. The semantic networks of the software are designed at different levels, in accordance with the decision tables and the relationship between the parameters for the diagnosis of the disease.
• Knowledge analysis: A rule-based method and MS diagnostic algorithm (Figure 3) are used to analyze the knowledge in the knowledge base. The production rules of the knowledge base are determined based on semantic networks and decision tables. The diagnosis algorithm is designed based on the 2004 MS Diagnosis Guideline and McDonald's 2017 diagnostic criteria and is approved by 10 neurologists. In each section of the diagnosis algorithm, a part of the production rules is fired.

### B. Designing the conceptual model with an object-oriented approach

To design the software database, the object-oriented methodology was developed using the IBM Rational Rose Enterprise v9. Operational, structural, and behavioral models of the software were created by drawing use cases, class-responsibility-collaboration (CRC), class, sequence, activity, state and collaboration diagrams.

3. Software development and implementation: SQL SERVER 2017 and Visual Studio 2017 v 15.8.9 and the C sharp programming language were used to codify the decision support software. Finally, the CDSS was deployed at the Neurology Department of the Shahid Beheshti Hospital of Kashan.
4. System evaluation: The software was evaluated in terms of efficiency and applicability. The efficiency of the CDSS for MS diagnosis was calculated using the real data recorded in 130 cases of patients admitted at the Shahid Beheshti Hospital of Kashan. Ninety-one patients with MS and 39 patients with stroke, neuromyelitis optica (NMO), brain and spinal cord tumors, and acute encephalomyelitis in the age range of 20 to 40 years between 2017 and 2019 were included based on sensitivity, specificity and accuracy with the use of IBM SPSS Statistics v25.0.

The receiver-operating characteristic (ROC) curve and surface area under the curve were also plotted to judge the performance and efficiency of the system. Also, the Kappa test was conducted, and its results were interpreted to evaluate the agreement of the physician's diagnosis and the proposed system's. The software applicability was evaluated by 10 neurologists based on the Nielsen standard questionnaire in three distinct areas, including the ease of learning, memorability, and satisfaction. The validity of the applicability questionnaire designed in this study was evaluated using content validity based on valid sources, and its reliability was determined by calculating Cronbach's alpha (α = 0.87).

## Results

The findings of this study are presented in 5 parts, as seen below.

### Results related to the requirements of users

The software input data were divided into five sections: demographic data, historical data, physical examinations, radiographic data, and laboratory data, which are described in Table 1.

**Table 1:** Necessary data of the clinical decision support for MS diagnosis.

**Range**	Class	Data components
Non clinical data	Demographic data	1. Age 2. Gender
	**Historical data**	1. Previous Neurological symtoms of the patient. 2. Suffering from viral diseases such as HIV. 3. Family medical history such as parents or sisters and brother.
Clinical data	Physical examination data	1. The number of clinical attack. 2. Attack duration. 3. Symptoms and signs of the disease: Color blind, gaze palsy, visual loss and blindness, diplopia, nystagemuse, inflation of the optic nerve, balance problem, tremor, weakness and paresis, spasticity, babensky, tic doulourux, Ihermitt, nausa, vertigo, pain, Bell’s palsy, fatigue, bowel problems, speech disorder, bladder dysfunction, sexual dysfunction, swallowing difficulties, paresetsia, numbness, sensory loss in face, cognitive impairment, epilepsy and sleep disorder and mental disorder, no fever or infection.
	**Radiographic data**	1. Type of plaque: T2 - hyper intense, T2 - Gallium enhancement and T2 Gallium non-enhancement. 2. Impairment place: brain ventricles, cortex areas, tentorial and spinal cord. 3. The number of plaques
	**Laboratory data**	1. Electrophoresis of cerebro spinal fluid: the number of oligo clonal bands (OCB) and amount of immunoglobulin G (IgG) index. 2. Visual Evoked potential (VEP): p100 delay duration of each and p100 amount difference between eyes.

The functional requirements of the system include simulation of the physician's decision-making process, thought and diagnosis, storing and creating a database of patients' data at each encounter, providing guidance and advice to the physician, providing reports required by physicians for decision making, presenting a summary of records to patients for them to find out the condition of their illness and back up their data to present to any neurologist.

Non-functional requirements of the system features include maintaining privacy and security of patients' data, tracking users' operations in software, the capability of maintaining and updating the system knowledge base based on new criteria of MS diagnosis, the capability of software extension, addition of new features and heightening its processing speed in the future.

### Results related to the design of the software knowledge base

Table 2 is the decision table for effective parameters in the diagnosis of MS, and Table 3 is the decision table for MS diagnosis based on its signs and symptoms. The inserted characters (++++, +++, ++ and +) at the intersection of the rows and columns in the decision tables show the extent of the effect of the decision parameter of the system on MS diagnosis. MS Diagnosis flowchart (Figure 3) includes a step-by-step diagnosis of MS by determining the degree of certainty.

**Table 2:** Decision table of diagnostic parameters of MS.

Diagnostic parameters	History of neurology signs and symptoms	One or more clinical attacks	Delay in nervous duration in visual evoked potential	Increasing IgG index and OCB in CSF	One or more plaque in MRI	History of viral infection	History of family	Lack of fever and infection	Age					Sex	
**Old**	Elderly	Young	Stripling	Infant	Female	Male
Multiple sclerosis (relapsing-remitting)	+ +	+ + + +	+ + +	+ + +	+ + + +	+ +	+ +	+ + +	+	+ +	+ + + +	+ + +	+	+ + + +	+ +

+ + + +: Symptoms that almost always exist (primary and prevalent symptoms) (75-100% of cases); + + +: Symptoms that usually exist (75-50% of cases); + +: Symptoms that exist in cases of disease progression (30-50% of cases); +: Symptoms that rarely exist (less than 30% of cases).

**Table 3:** Decision table of signs and symptoms of MS.f MS.

Signs and symptoms /Place of plaques in CNS	Sleep disorder	Cognitive impairment	Epilepsy	Mental disorder	Facial sensory loss	Numbness	Paresthesia	Swallowing difficulties	Sexual dysfunction	Bladder dysfunction	Fatigue	Speech disorder	Bell’s palsy	Pain	Bowel problems	Vertigo	Nausea	Lhermitte	Tic Doulourux	Babinski	Spasticity	Weakness	Tremor	Balance problem	Nystagmus	Inflammation of the optic nerve	Diplopia	Visual loss and blindness	Gaze palsy	Color blind
**Optic nerves**																										+		+ + +		+ + +
**Cerebellum**												+ + +				+ +							+ + +	+ + +						
**Brain**	+ +	+ + +	+ +	+ + +							+ + + +									+ + + +	+ + +									
**Brain stem**	+ +				+ + +	+ + +	+ + + +	+ +				+ + +	+ +	+ + +	+ +	+ +	+ +	+ +	+ + +					+ + +	+ +		+ + +		+ +	
**Spinal cord**	+ +					+ + +	+ + + +		+ +	+ + +								+ +		+ + + +	+ + +	+ + + +								

+ + + +: Symptoms that almost always exist (primary and prevalent symptoms) (75-100% of cases); + + +: Symptoms that usually exist (75-50% of cases); + +: Symptoms that exist in cases of disease progression (30-50% of cases); +: Symptoms that rarely exist (less than 30% of cases).

### Results related to the design of the object-oriented conceptual model of the software

**Figure 4.**
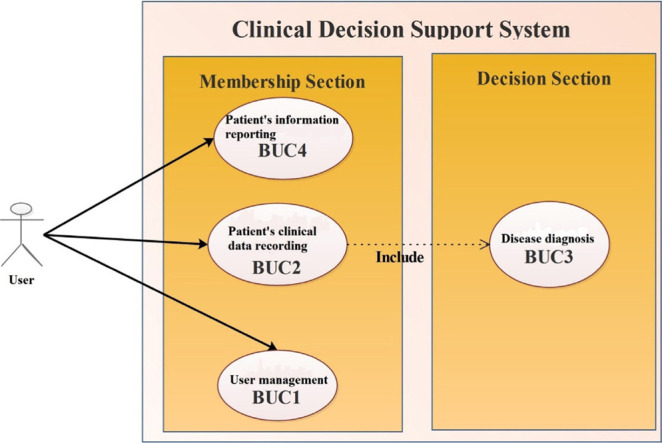
Use-case diagram of the clinical decision support system for MS diagnosis.

**Figure 5.**
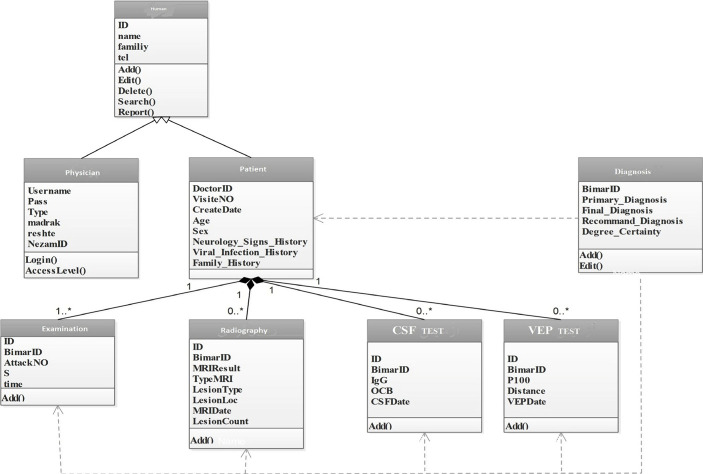
Class diagram of the clinical decision support system for MS diagnosis.

Figure 4 illustrates the use-case diagram and main functions of the system, each of which consists of sub-items. The MS diagnostic decision support software has classes of human, physician, patient, examination, CSF testing, visually evoked potential (VEP) testing and radiography, as shown in Figure 5.

### Result related to software development

**Figure 6:**
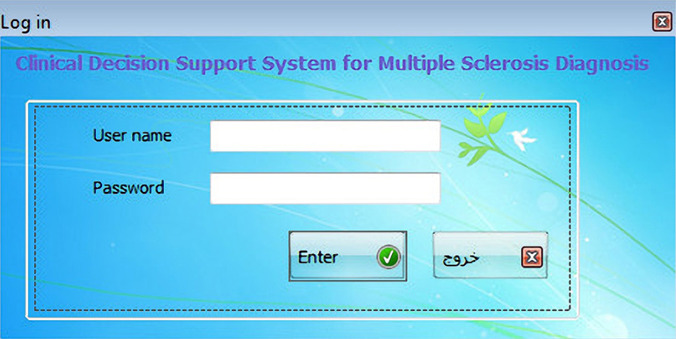
Log-in page of software users.

**Figure 7:**
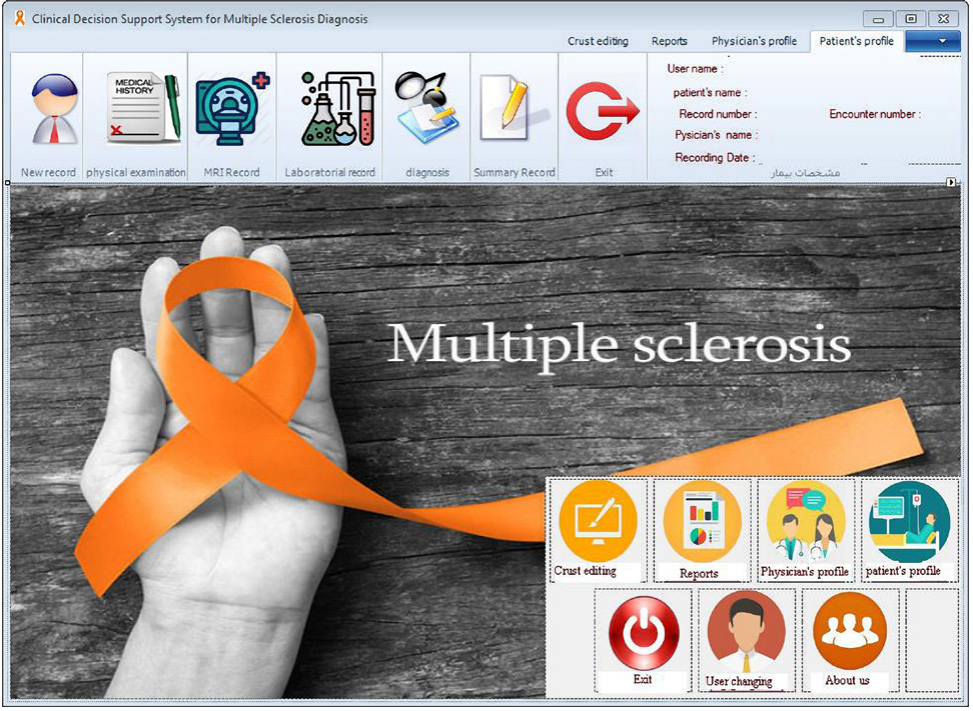
Main page and background of the clinical decision support system.

**Figure 8:**
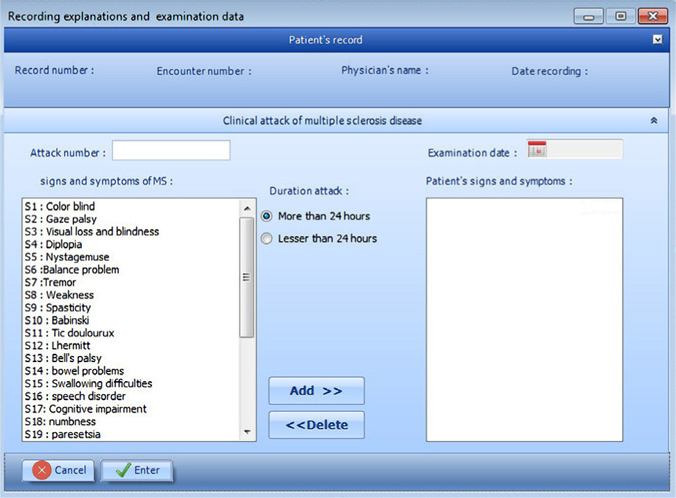
Patient profile, explanation and physical examination page.

**Figure 9:**
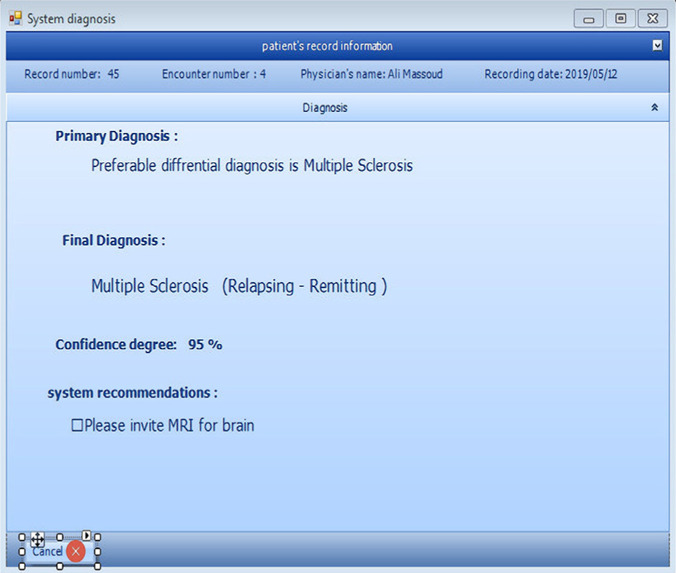
System diagnosis page.

The software includes the main sections of the patient profile, physician profiles, and reports. Patient profiles incorporate sub-parts for medical recording, recording explanations and examination data, recording MRI data, recording laboratory data, diagnosis, and a summary of the records. Physicians are able to edit their information on their profile and their patient's medical record. They can also provide their reports and information in the software reporting section. The system output is the diagnosis of MS with a degree of certainty as well as the diagnosis of other diseases and suggestions for the examination of the differential diagnosis of MS. The software developed in this study provides the output and new advice to the physician at each encounter of the patient based on newly recorded data in the system. The system also provides users with the capability of describing their responses and reasons. Figures 6-9 show parts of the software user interface.

### Results related to the evaluation, efficiency and applicability of the software

**Figure 10:**
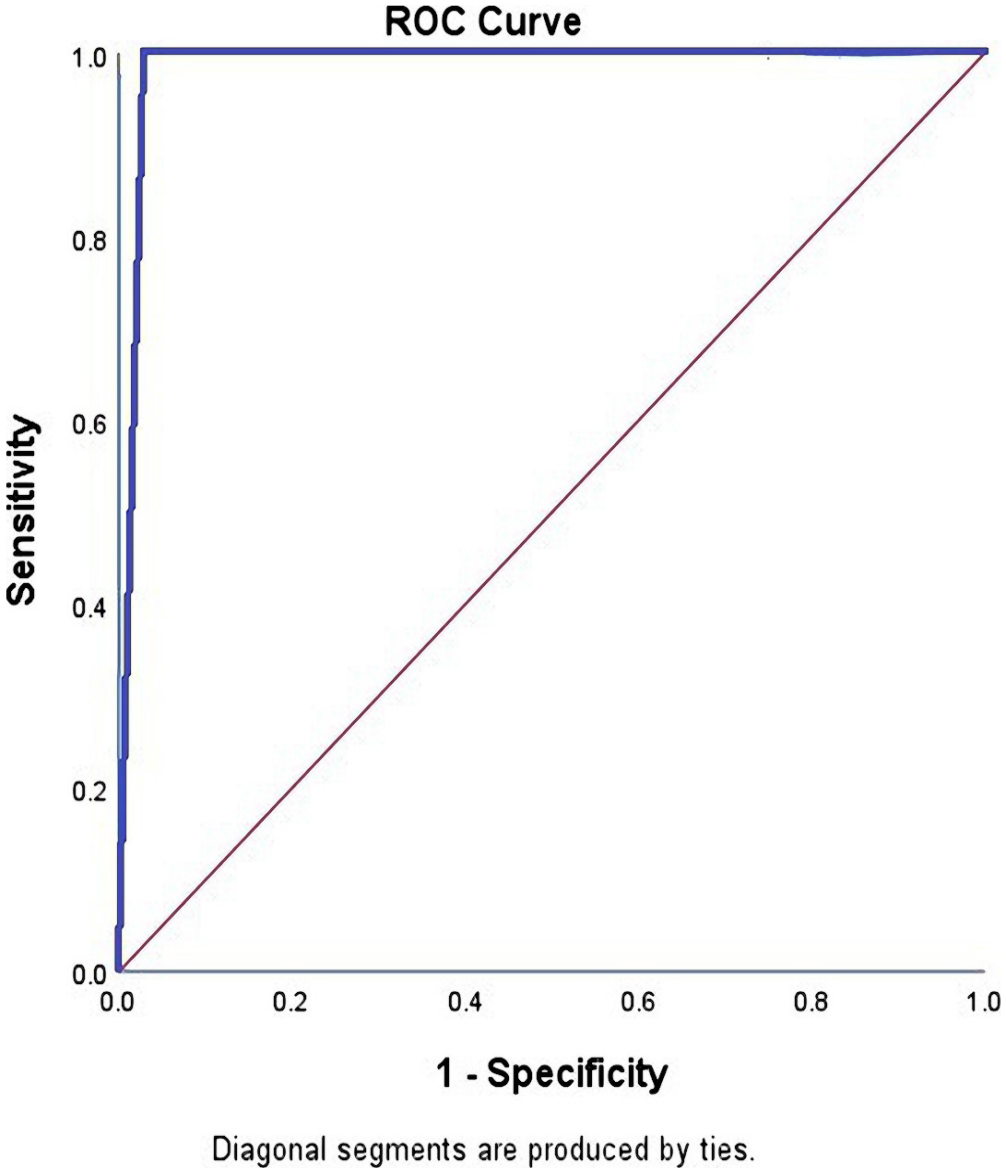
Log-in page of software users.

The decision support software developed in this paper has a sensitivity of 100%, a specificity of 97%, an accuracy of 99%, and a surface area under the ROC curve of 0.987 (Figure 10). Also, the software had the same diagnosis as the physician in 129 cases, had a different diagnosis in 1 case, and the value of the k variable in the Kappa test for the system equaled 0.98 with an error rate of 0.0001. Therefore, according to Landis's interpretation table (Table 4), the diagnosis of the decision support system in this study was perfectly consistent with the final diagnosis of the neurologist.

**Table 4:** Interpretation of the Kappa Test results.

k	Interpretation
**<0**	No agreement
**0.0-0.20**	Slight agreement
**0.21-0.40**	Fair agreement
**0.41-0.60**	Moderate agreement
**0.61-0.80**	Substantial agreement
**0.81-1**	Almost perfect agreement

The average score of software users was 98.33%, 96.65%, and 96.9%, regarding ease of learning, memorability, and satisfaction, respectively. Therefore, the applicability of the CDS software for MS diagnosis was confirmed by the neurologists.

## Discussion

The results of the software evaluation revealed that there was a complete consistency between the system diagnosis and the final diagnosis recorded in the patient records, and also reflected the high acceptability of the system and the satisfaction of all users' operational expectations, as well as the system's applicability in health environments.

All the diagnostic parameters affected the efficiency and performance of software diagnosis. In this paper, 45 parameters presented in Table 1 were used by the system. Ahmad al-Hajji, an Arabian researcher in computer science, developed a rule-based expert system for the diagnosis of neurological diseases, which had used four clinical signs and symptoms for MS diagnosis. Its diagnostic accuracy was reported to be 0.8 [35]. A diagnostic system for neurodegenerative disorders has been used only for 5 signs of memory impairment, lack of muscle movement control, urinary tract disorders, and fatigue for MS diagnosis and diagnosed 50 patients accurately [36]. The system of diagnosis of neurological disorders, in addition to 73 signs and symptoms, used age and gender parameters, which were not evaluated [39]. The neurology diagnosis system project uses a combination of signs and symptoms of the disease, the results of various laboratory tests, and the results of MRI and CT scan imaging, and its evaluation result has not been reported [38].

A powerful knowledge base is one of the strengths of CDSSs because it makes its inference engine optimal, smart, and more error-tolerant. According to the studies, decision tables, decision trees, and semantic networks are helpful in designing a knowledge base for decision support and expert systems and represent the link between signs and symptoms and diseases, and the rules of production are derived from them. The expert system for the diagnosis of neuromuscular disorders has used the decision tree, and the neurological expert system has used the decision table and the decision tree. The decision table has been used in the design of the knowledge base of the diagnostic system for nervous demyelination diseases and neurological disorders. The Fuzzy Expert System for MS diagnosis in Italy used the graphic diagram of the semantic network to organize and represent the knowledge base. In this paper, the approaches to the decision table and the semantic network are used to represent the production rules.

Using a suitable reasoning method and technique to diagnose in decision support systems is important in the efficiency of the software. A rule-based method is one of the most widely used methods to diagnose diseases, especially MS [40]. This method manages well-defined issues with text-based knowledge. The web-based decision support system has used production rules with a sensitivity of 93% and a specificity of 86.6% [39]. Ahmad al-Hajji developed a rule-based diagnostic system of nervous diseases with an accuracy of 0.8 [35]. In 2009, Linder *et al.* used the computer-assisted diagnostic system for the differential diagnosis of MS using logistic regression and artificial neural networks in Germany. The sensitivity and accuracy of the statistical method in the classification of patients were 0.84 and 0.54, respectively, and the sensitivity and accuracy for the artificial neural network in the classification of MS patients from other patients were 0.95 and 0.83, respectively [41]. The differential diagnostic system of MS with relapsing-remitting clinical trend had an accuracy of 0.87 and AUC of 0.75 using statistical methods [42]. The fuzzy decision system for MS diagnosis using the Sogno Fuzzy Model has a sensitivity and specificity of 0.87 and 0.75 for MS diagnosis, respectively, and its area Under the ROC curve was reported to be0.85 [43]. The artificial neural network method with multiple neural networks in MS diagnosis had the highest efficiency compared to other rule-based methods [44, 45].

The use of an object-oriented conceptual model helps software analysts to design quick and logical systems in the changing conditions of the business atmosphere, enhancing the quality of analysis of the users' requirements and software design documentation. The system developed in this paper and the Neurology Diagnose System Project at the University of Tryp Habana used object-oriented design and UML modeling language diagrams, which are deemed to be strengths of the software and show the operational, structural, and behavioral perspectives of the software.

In order to access the proposed system, we encountered limitations such as the limited number of medical records of patients with NMO and ADEM, incomplete data records in the medical records of patients, and their removal in the evaluation stage.

Given that a rule-based method has limited flexibility, it is suggested that a combination of rule-based and case-based methods be used to overcome this problem in decision support systems. Also, to overcome the uncertainty problems in medicine, and the different parameters of MS diagnosis and their values in different patients, a combination of rule-based and fuzzy logic methods should be used. The proposed decision system in this study could be expanded for the diagnosis of other types of MS phenotypes and for their management and treatment.

## Conclusions

The results of the evaluations of the efficiency and applicability of the software demonstrate the high acceptability of the system and satisfaction of all the operational expectations of users, and that CDSS provides guidance in decision-making and helps physicians in the timely and accurate diagnosis of MS. These are the results of proper knowledge engineering and the use of effective parameters in the diagnosis, analysis process and object-oriented conceptual design of the software, the use of appropriate reasoning methods, and the development of MS diagnosis algorithm based on the latest McDonald's MS diagnosis criteria and guideline, as well as the centrality of final users and neurologists in the design of the graphical user interface of the system.

The rule-based method is one of the most widely used reasoning methods for the diagnosis of MS having proper efficiency and benefits such as modularity and general combination and description of the results. Being understood and well accepted by physicians, this method manages well-defined issues with text-based knowledge. In CDSSs and expert systems, a combination of different reasoning methods can be used based on the system's objective in order to enhance the efficiency and intelligence of the system.

## Conflict of Interest

The authors declare that there is no conflict of interest.
